# Differences in Health and Health Behaviors Between State Employees and Other Employed Adults in Oregon, 2007

**Published:** 2010-08-15

**Authors:** Ying Han, Daniel S. Morris, Stacey Schubert, Duyen Ngo, Jane M. Moore

**Affiliations:** Oregon Public Health Division, Portland, Oregon; Health Promotion and Chronic Disease Prevention, Oregon Public Health Division; Oregon Public Health Division, Portland, Oregon; Oregon Public Health Division, Portland, Oregon; Oregon Public Health Division, Portland, Oregon

## Abstract

**Introduction:**

Worksite health promotion and interventions have gained popularity among state agencies. We studied the health behaviors and health characteristics of adults employed in state agencies in Oregon and compared those state employees with the statewide population of employed, insured adults.

**Methods:**

We used data from the Oregon Behavioral Risk Factor Surveillance System (BRFSS) and a modified BRFSS survey administered to state employees. State employees were compared with employed, insured BRFSS respondents in total and then separately for men and women.

**Results:**

The prevalence of healthy weight was lower among state employees compared with the statewide population of employed, insured adults (29% vs 35%), and the prevalence of obesity was higher (35% vs 26%). State employees were also less likely to meet physical activity recommendations (44% vs 56%). Diabetes prevalence was higher among state employees (7% vs 5%), and self-reported excellent or very good health status was lower (54% vs 64%).

**Conclusions:**

State employees differ from the statewide population of employed, insured adults on a number of health behaviors and conditions. These differences suggest obesity prevention and diabetes control as priority areas for state agency worksite interventions.

## Introduction

US employers pay higher health insurance costs in part because of the increasing demand for health care services related to chronic diseases. Since 2000, premiums for employer-sponsored family health care insurance have increased by 87% (1). Tobacco use, physical inactivity, and poor nutrition are common, modifiable behaviors that contribute to chronic diseases (2). Depression and other mental illnesses are also associated with higher medical costs and chronic diseases (3). Medical care for people with chronic conditions in the United States accounts for more than 75% of the nation's $2 trillion in medical care expenditures (4).

Government agencies are among the largest employers in the United States (5). In 2008, 48,200 people were employed by the state of Oregon's noneducational agencies that are covered by Public Employees' Benefit Board (PEBB) (6). Public employees are more likely than employees in the private sector to receive health insurance (7), and almost all state employees in Oregon are eligible for health insurance benefits from PEBB (8). However, little is known about the differences between public- and private-sector employees in the health characteristics and health behaviors associated with higher employer costs.

We used data from the Oregon Behavioral Risk Factor Surveillance System (BRFSS) and a modified BRFSS survey administered to state employees to describe health behaviors and health characteristics of adults employed in state agencies and to compare them with employed, insured adults statewide. We sought to identify health disparities borne by state employees and to provide guidance for worksite health promotion efforts in state agencies.

## Methods

### Oregon Behavioral Risk Factor Surveillance System

BRFSS is a state-based, random-digit-dialed telephone survey of the noninstitutionalized population aged 18 years or older and is used to track health conditions and risk behaviors. BRFSS is exempt from full review by the Centers for Disease Control and Prevention institutional review board. We used Oregon BRFSS data from 2007 for this study and weighted the data to correct for nonresponse and noncoverage errors (9). The response rate of the 2007 Oregon BRFSS was 47%; of those responses, 35% met our study's inclusion criteria (3,556 completed surveys).

### Oregon BRFSS Survey of State Employees

We developed the BRFSS Survey of State Employees (BSSE) to collect information on health conditions and risk behaviors of Oregon state employees. Created by modifying the BRFSS survey, the BSSE was conducted in 2005 and 2007 with support from the Oregon Public Health Division of the Department of Human Services (DHS) and PEBB. The BSSE protocol was reviewed by a committee at the Public Health Division and deemed a public health surveillance activity that was not subject to institutional review. For our study, we used 2007 BSSE data. Before the survey, agency directors notified state employees by e-mail regarding this survey. The same contractor who conducted the Oregon BRFSS survey also conducted the BSSE telephone interviews. Work telephone numbers were used to make initial contact, and interviewers arranged to conduct the interview at another time or location if that was preferred. In 2007, the response rate for state employees was 59% (1,633 completed surveys).

### Sample

We included only BRFSS respondents who reported being "employed for wages" and who reported having "any kind of health care coverage, including health insurance, prepaid plans such as HMOs [health maintenance organizations], or government plans such as Medicare." Nearly all state employees have medical insurance through PEBB; only 4% opted out of medical insurance. State employees must receive coverage through another source to opt out of medical insurance through PEBB, so all BSSE respondents had health insurance.

We refer to employees working at state agencies as state employees. Because demographic characteristics are different between state agency employees and university employees (10), we excluded university system employees from this analysis. Our data and analysis do not include employees without a telephone at work. We used a random sample of 6,024 state employee telephone numbers from human resources records for the BSSE survey. We oversampled DHS employees to increase the reliability of future analysis and research for that subgroup of employees, who have been a pilot group for the Oregon Healthy Worksite Initiative (10). DHS employees made up 80% of the sampling frame but account for 27% of the employees among all the state agencies. The final sample included 4,819 employee telephone numbers from DHS and 1,305 employee telephone numbers from Department of Employment, Department of Administrative Services, Department of Transportation, Oregon State Police, Department of Corrections, and 80 other state agencies. The sample contained a larger proportion (71%) of women than the actual percentage of women (54%) in the state agency payroll system because the oversampled DHS employs a higher proportion of women than other state agencies. To improve the accuracy of our estimates, we applied a poststratification weight based on sex.

### Primary study outcome measures

BSSE questions on demographics, smoking, health status, and health conditions were identical to BRFSS questions. To create questions on physical activity and nutrition in BSSE, we used the same criteria as the core BRFSS questions. From the responses, we calculated whether respondents met CDC physical activity recommendations and ate 5 or more servings of fruits and vegetables per day based on *Dietary Guidelines for Americans, 2005* (11).

In both surveys, calculations of body mass index (BMI) (weight in kilograms divided by height in meters squared) were based on self-reported height and weight and grouped into 4 standard categories: underweight, less than 18.5 kg/m^2^; normal weight, 18.5 to 24.9 kg/m^2^; overweight, 25.0 to 29.9 kg/m^2^; and obese, 30.0 kg/m^2^ or higher. Respondents were asked to rate their general health on a scale from excellent to poor. We dichotomized health status into excellent or very good and others. For depression, respondents were asked whether they had been told by a doctor or other health professional that they had depression in the past 12 months. Hypertension was similarly assessed by asking whether respondents were told by a health care professional that their blood pressure was high. Hypertension during pregnancy, prehypertension, and borderline hypertension were not included in our analysis. Cholesterol screening was based on an affirmative answer to whether respondents had had their cholesterol checked during the preceding 5 years. High blood cholesterol was defined as respondents' having been told by a health care professional that their blood cholesterol was high. Having asthma was defined as respondents having ever been told by a doctor, nurse, or other health care professional that they had asthma and still had asthma at the time of survey. Having diabetes was defined as respondents'  having ever been told by a doctor that they had diabetes. Gestational diabetes, prediabetes, and borderline diabetes were not included in the analysis. Similarly, having arthritis was defined as having ever been told by a doctor or other health professional that they had some form of arthritis, rheumatoid arthritis, gout, lupus, or fibromyalgia.

### Data analysis

We used Stata 9.2 (StataCorp LP, College Station, Texas) for data analysis. Missing values in each survey were excluded from analysis. We age-adjusted data from both the 2007 Oregon BRFSS and the 2007 BSSE to the 2000 standard population in 3 age categories: 18 to 34 years, 35 to 54 years, and 55 years or older. We calculated point estimates and confidence intervals of BRFSS respondents based on Taylor linearized variance estimation according to the BRFSS complex survey design (12). We produced estimates of BSSE responses based on normal approximation of the binomial distribution (13). We compared point estimates for state employees and statewide employed, insured adults and for men and women separately. To test for significance between BSSE and BRFSS point estimates, we looked for overlap in the 95% confidence intervals. This method yields a conservative assessment of significant differences (14-16), under the assumption that the BSSE respondents and the BRFSS respondents did not overlap.

## Results

More women and college graduates worked at state agencies than among the statewide employed, insured population (Table 1). The mean age of state employees was higher than that of the statewide employed, insured population. Among state employees, more men than women had an annual household income of at least $50,000 and were a college graduate.

**Figure 1:**
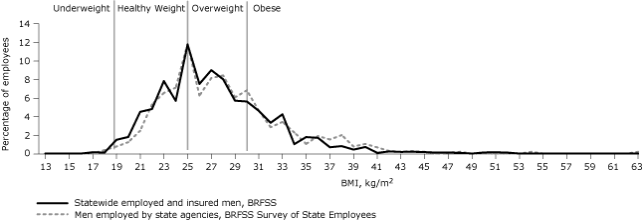
Proportion of employed, insured men statewide and men employed by state agencies who were classified by body mass index (BMI) as underweight, healthy weight, overweight, and obese, Oregon, 2007. Statewide data are from the Behavioral Risk Factor Surveillance System (BRFSS) for Oregon, and data for state agency employees are from the BRFSS Survey of State Employees.

**Figure 2:**
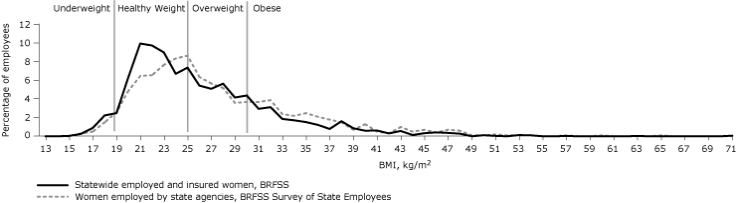
Proportion of employed, insured women statewide and women employed by state agencies who were classified by body mass index (BMI) as underweight, healthy weight, overweight, and obese, Oregon, 2007. Statewide data are from the Behavioral Risk Factor Surveillance System (BRFSS) for Oregon, and data for state agency employees are from the BRFSS Survey of State Employees.

A lower percentage of state employees had a healthy weight compared with the statewide employed, insured population. The percentage of obesity was also higher, which was also seen among men and women separately. Although the BMI distribution was similar among men in both the state employee group and the statewide employed, insured population (Figure 1), among women the BMI distribution showed that state employees were heavier than the comparable group of employed, insured women (Figure 2).

**Table:** 

The range for healthy weight is BMI 18.5-24.9 kg/m^2^, and the range for overweight is BMI 25.0-29.9 kg/m^2^. Reported BMIs ranged from 17 to 63 kg/m^2^. The distribution of BMI was similar for both groups. The largest proportion of men, 12%, had a BMI of 25 kg/m^2^. Approximately 1% had a BMI of 18.5 kg/m^2^. Approximately 6% of employed, insured men statewide and 7% of men employed by state agencies had a BMI of 30 kg/m^2^,limit for the obese range.

**Table:** 

The range for healthy weight is BMI 18.5-24.9 kg/m^2^, and the range for overweight is BMI 25.0-29.9 kg/m^2^. Reported BMIs ranged from 15 to 71 kg/m^2^. The largest proportion of employed, insured women statewide, 10%, had a BMI of 21 kg/m^2^, and the largest proportion of women employed by state agencies, 9%, had a BMI of 25 kg/m^2^. Approximately 2% of employed, insured women statewide and of women employed by state agencies had a BMI of 18.5 kg/m^2^. Approximately 7% of employed, insured women statewide had a BMI of 25 kg/m^2^, as do approximately 9% of women employed by state agencies. Approximately 4% of both groups had a BMI of 30 kg/m^2^, the lower limit for the obese range.

The prevalence of meeting CDC physical activity recommendations was lower among both men and women working in state agencies (Table 1). Although women in the state employed, insured population ate more fruits and vegetables than did men, overall fruit and vegetable consumption was low across all subpopulations in this study.

The prevalence of self-reported excellent or very good health status was lower among men and women in the state employee population than among their counterparts in the statewide employed, insured population (Table 2). Clinical diagnosis of depression in the preceding year was more common among female state employees than male state employees. State employees had a higher 5-year cholesterol screening rate than statewide employed, insured adults, and more state employees than the statewide employed, insured population took medicine if they had hypertension. Diabetes was more common among state employees than statewide employed, insured adults.

## Discussion

Our study findings indicate that having access to health benefits does not guarantee employees' good health. The prevalence of obesity was higher among state employees than among employed, insured adults statewide, and meeting recommendations for physical activity was lower. Because of these disparities, state agencies may bear higher obesity-related costs in productivity loss, absenteeism, and health care coverage than other employers. Because the proportion of state employees who reported being in excellent or very good health was lower and the prevalence of diabetes was higher than among employed, insured adults statewide, the state may already be paying higher health care costs for its employees.

Although the literature regarding health characteristics of state employees is scarce, our findings agree with those from wellness promotion efforts in other states (17). Surveys such as the BSSE allow for meaningful comparisons between private- and public-sector employees and can inform changes to worksite health promotion policies and employee benefits at state agencies. Results from the BSSE have led to worksite wellness programs and policies such as a Weight Watchers benefit for state employees. In the first 3 months that the benefit was offered (January-March 2009), 1,588 employees began attending classes at their worksite, 1,763 got vouchers for meetings outside of work, and another 1,254 signed up for an online membership. The at-work members lost nearly 18,000 pounds in the first 3 months of the program (18). Subsequent BSSE surveys will be used to monitor the effect of these policies on employee wellness.

The household income gap observed between women and men employed by the state may reflect lower individual earnings for women (19). Our findings that overall state employees' income was close to that of the statewide employed, insured population is consistent with information reported elsewhere (20). However, women who work in state agencies had lower income than women in the statewide employed, insured population, whereas the income of men employed in state agencies did not differ significantly from income of the statewide employed, insured population. Previous studies found the inverse relationship between socioeconomic status and obesity to be stronger among women than men (21). In our study, lower income among women employed by the state may explain the higher obesity rate.

Our finding of a higher prevalence of 1-year diagnosed depression among women than men in our study is in line with findings from other studies (22). Although the difference in depression prevalence may be partially explained by sex-related differences in cognitive styles, certain biologic factors, and economic stresses (23), it could also reflect the different medical care use between men and women. Our observation of a higher prevalence of hypertension in men than women employed by the state is consistent with an earlier finding that hypertension was more prevalent among men than women at a sample of workplaces (24). The higher cholesterol screening rate in state employees than among the statewide employed, insured population is probably the result of PEBB's offering free cholesterol screenings at worksites.

We note several limitations in this study. First, we rely on self-reported data in both surveys. Assessing the influence of recall bias and interview bias on our results is difficult, but these biases should not be different between these 2 surveys. Second, both BRFSS and BSSE had moderate response rates, but the rates were similar to the national median BRFSS response rate (25). Third, we were not able to compare other factors such as race, ethnicity, or job characteristics between the 2 populations. These factors may predict both people's decisions regarding working in the public or private sector and their health. Oregon's population is predominantly white, and we did not have the sample size to conduct subgroup analyses by race or ethnicity. Fourth, we cannot exhaustively compare the detailed health benefits received by state employees and other workers across the state. Different quality of care may partially explain some of the disparities in diagnosed health conditions between state employees and other employed, insured adults in the state. Although not all state employees have chosen to be covered by the state plan, those who are not covered must receive coverage through another source to opt out of the state plan. Therefore, we do not believe that the BSSE's undercoverage of state employees who opted out of the health insurance plan has a substantial effect on our findings. Finally, our use of Oregon-specific data limits the generalizability of our findings. Other states may consider their own surveys of state employees to obtain relevant data for intervention planning and evaluation.

State employees differ from the statewide population of employed, insured adults on a number of key health behaviors and conditions. These differences suggest obesity prevention and diabetes control as priority areas for worksite interventions for state employees. Worksite health promotion and intervention planning would benefit from future studies that explore job characteristics and the effect of socioeconomic status on health behaviors or conditions among state employees. Continued surveillance of state employees will be necessary to target and evaluate worksite wellness interventions.

## Figures and Tables

**Table 1:** Comparison of Demographic Characteristics and Health Risk Factors Between Oregon State Employees and Oregon General Employed Adults With Health Insurance, Oregon Behavioral Risk Factor Surveillance System (BRFSS) and BRFSS Survey of State Employees, 2007^a^

**Characteristic**	**State Employees, n = 1,633**	**General Employed Insured Adults, n = 3,556**	**State Employees, Male, n = 474**	**General Employed Insured Adults, Male, n = 1,458**	**State Employees, Female, n = 1,159**	**General Employed Insured Adults, Female, n = 2,098**
**Mean age, y (range)**	47 (19-73)	42 (18-88)	48 (19-73)	41 (18-86)	47 (19-73)	42 (18-88)
**Female, % (95% CI)**	55 (52-58)	46 (44-48)	NA	NA	NA	NA
**Annual household income ≥$50,000, % (95% CI)**	58 (55-61)	60 (58-62)	64 (60-69)	62 (59-65)	52 (49-56)	58 (56-60)
**College graduate, % (95% CI)**	57 (54-60)	44 (41-47)	67 (62-71)	42 (38-46)	49 (46-52)	46 (42-49)
**Current smoker, % (95% CI)**	11 (10-13)	14 (12-15)	10 (6-13)	14 (12-17)	13 (11-15)	13 (12-15)
**Meeting physical activity recommendations,^b^ % (95% CI)**	44 (41-47)	56 (53-59)	47 (42-52)	57 (53-62)	41 (38-44)	55 (52-59)
**Eating ≥5 servings of fruits and vegetables every day, % (95% CI)**	23 (21-25)	25 (23-28)	20 (16-24)	22 (18-26)	25 (22-28)	29 (26-32)
**Body mass index (kg/m^2^), % (95% CI)**						
Healthy weight, 18.5-24.9	29 (26-32)	35 (34-37)	23 (18-27)	27 (25-30)	35 (32-38)	45 (43-48)
Overweight, 25.0-29.9	35 (32-37)	37 (35-39)	41 (36-46)	45 (42-48)	29 (26-32)	28 (26-31)
Obese, ≥30.0	35 (32-38)	26 (25-28)	36 (32-41)	28 (25-31)	34 (31-37)	25 (23-27)

Abbreviations: CI, confidence interval; NA, not applicable.

a Data were age-adjusted, except for mean age.

b Recommendations from *Dietary Guidelines for Americans, 2005* (11) were used to define whether physical activity recommendations were met.

**Table 2:** Prevalence of Health Conditions and Behaviors Among Oregon State Employees and Oregon General Employed Adults With Health Insurance, Oregon Behavioral Risk Factor Surveillance System (BRFSS) and BRFSS Survey of State Employees, 2007^a^

**Characteristic**	**State Employees, All (**n = 1,633), % (95% CI)	**General Employed Insured Adults, All (**n = 3,556), % (95% CI)	**State Employees, Male (**n = 474), % (95% CI)	**General Employed Insured Adults, Male (**n = 1,458), % (95% CI)	**State Employees, Female (**n = 1,159), % (95% CI)	**General Employed Insured Adults, Female (**n = 2,098), % (95% CI)
Reporting excellent or very good health status	54 (51-57)	64 (62-66)	53 (48-58)	63 (60-66)	54 (51-57)	66 (64-68)
Clinically diagnosed depression, past 12 months	12 (11-14)	9 (7-12)	7 (5-9)	7 (4-12)	17 (14-19)	12 (9-17)
Hypertension	24 (22-26)	23 (20-25)	28 (24-32)	26 (23-30)	21 (18-23)	19 (17-21)
Taking hypertension medication currently, if have hypertension	68 (60-76)	54 (48-60)	68 (56-79)	51 (44-58)	68 (58-78)	62 (50-73)
Cholesterol checked within past 5 years	82 (79-84)	74 (72-77)	79 (74-83)	73 (69-76)	84 (82-86)	76 (73-79)
High blood cholesterol	31 (28-34)	31 (28-34)	34 (28-39)	35 (31-39)	29 (26-32)	27 (24-30)
Diabetes	7 (6-9)	5 (4-6)	7 (5-9)	5 (4-7)	8 (6-9)	4 (3-5)
Asthma	9 (8-11)	9 (8-10)	8 (5-10)	7 (5-8)	11 (9-13)	11 (10-13)
Arthritis	23 (21-25)	21 (19-23)	21 (17-24)	18 (15-21)	25 (23-28)	24 (22-27)

Abbreviation: CI, confidence interval

a Data were age-adjusted.
